# Involvement of the Cerebral Monoamine Neurotransmitters System in Antidepressant-Like Effects of a Chinese Herbal Decoction, Baihe Dihuang Tang, in Mice Model

**DOI:** 10.1155/2012/419257

**Published:** 2012-08-23

**Authors:** Meng-Li Chen, Jie Gao, Xin-Rong He, Qian Chen

**Affiliations:** ^1^Department of Clinical Pharmacology, General Hospital of Peoples Liberation Army, Beijing 100853, China; ^2^Department of Pathology, General Hospital of Peoples Liberation Army, Beijing 100853, China; ^3^Traditional Chinese Medicine Dispensary, General Hospital of Peoples Liberation Army, Beijing 100853, China

## Abstract

Baihe Dihuang Tang (BDT) is a renowned Chinese herbal formula which is commonly used for treating patients with mental instability, absentmindedness, insomnia, deficient dysphoria, and other psychological diseases. These major symptoms closely associated with the depressive disorders. BDT was widely popular use for treating emotion-thought disorders for many years in China. In the present study, the antidepressant-like effect of BDT in mice was investigated by using the forced swim test (FST) and the tail suspension test (TST). The underlying mechanism was explored by determining the effect of BDT on the level of cerebral monoamine neurotransmitters. BDT (9 and 18 g/kg, p.o. for 14 days) administration significantly reduced the immobility time in both the FST and the TST without changing locomotion in the open field-test (OFT). Moreover, BDT treatment at the dose of 18 g/kg inhibited reserpine-induced ptosis. Meanwhile, BDT enhanced 5-HT and NA levels in mouse cerebrum as well as decreased the ratio of 5-HT compared to its metabolite, 5-HIAA, (turnover, 5-HIAA/5-HT) after TST. The results demonstrated that the antidepressant-like effect of BDT is mediated, at least partially, via the central monoaminergic neurotransmitter system.

## 1. Introduction

Depression is a mental illness that significantly affect a person's thoughts, behavior, feelings, and physical well-being and has become a major global psychiatric problem. In whole globe, approximately 450 million people suffer from depression or behavioral disorder. According to prediction, depression will become the second common disease by the year 2020 [1]. The classical antidepressants include the tricyclic antidepressant (TCA), monoamine oxidase inhibitor (MAOI), selective serotonin reuptake inhibitor (SSRI), noradrenergic reuptake inhibitor (NARI), and serotonin and noradrenaline reuptake inhibitor (SNRI) [2, 3]. Although these drugs show excellent efficacy, most of them frequently produce undesirable adverse effects. So it is urgent to explore more promising antidepressants for clinical needs of depressed patients.

Traditional herbal formulae have been clinically used for thousands of years in China. Nowadays, the use of traditional herbal formulae has provided us a prospective alternative in the treatment of depression [4, 5]. Baihe Dihuang Tang (BDT) is a renowned Chinese herbal formula and firstly described in “synopsis of the Golden Chamber” (Jinkui Yaolue) written by Zhang Zhong Jing in the early 3th century. It is composed of two component herbs: lily bulb (Bulbus Lilii) and rehmannia root (Radix Rehmanniae). BDT is commonly used in folk for the therapeutic treatment of mental instability, absentmindedness, insomnia, deficient dysphoria, and other psychological diseases [6]. These major symptoms closely associated with the depressive disorders. BDT is widely popular use for treating emotion-thought disorders for many years in China. Some clinical studies have demonstrated antidepressant-like effects of BDT[7, 8]. Recently, pharmacological studies also have authenticated that plants of the BDT and some of their chemical constituents, including saponins, iridoids, and polysaccharides, displaying nervous system activities. Prepared *Rehmannia*, steamed roots of *Rehmannia glutinosa*, have effects on depression-like disorders, and antioxidation may be one of the mechanisms underlying its antidepressant action [9]. Catalpol, an iridoid glycoside, contained richly in *Rehmannia*, is found to be neuroprotective effect antioxidative ability, reduces cognitive impairment significantly [10–12] and therapeutic potential against inflammation-related neurodegenerative diseases [13]. As the component herbal drug, lily bulb or saponins from lily bulb also have depressant-like effects involved in the serotonergic system [14, 15] and the hypothalamic-pituitary-adrenal (HPA) axis in animal [16].

In the present study, we aim to investigate the antidepressant-like effects of BDT by using the forced swim and tail suspension tests in mice. The underlying mechanism of antidepression is explored by measuring the levels of monoamine neurotransmitters in mouse cerebrum.

## 2. Materials and Methods

### 2.1. Chemicals and Reagents

Desipramine, norepinephrine (NE), dopamine (DA), serotonin (5-HT), 8-O-acetylharpagide, and 5-hydroxyindoleacetic acid (5-HIAA) were obtained from Sigma-Aldrich (St. Louis, MO, USA). Reserpine injection (1 mg/mL) was produced by Guangdong Bangmin Pharmaceutical Co., Ltd. Ginsenoside Re, quercetin, was supplied by the State Drug Analysis Institute (Beijing, China). All other reagents and solvents used in the study were of analytical grade.

### 2.2. Plant Materials and Preparation of BDT

Bulbus Lilii (BL) and Radix Rehmanniae (RR) were purchased from Tongrentang Chinese Pharmaceutical Co. Ltd. (Beijing, China). The two herbs were ground into a coarse powder, respectively. BDT was formulated by mixing the two herbal powders in relative proportions according to a ratio of 2 : 1 (BL : RR). The herbal powder mixture was boiled in 8 volumes of water (v/w) in reflux for 60 minutes. The extraction procedure was repeated twice for 45 minutes. The pooled extract was filtered to remove debris. The concentrated extract was then dried by lyophilization to obtain the extract at a yield of 32.84% (w/w). The extract was stored in the desiccator at 4°C until use. Contents of total saponins [17], total flavonoids [18], total iridoids [19], and total polysaccharides [20] in BDT extract were measured by modified methods, using ginsenoside Re, quercetin, 8-acetylharpagide, and dextran as standards, respectively. The results indicated that BDT contained saponins, flavonoids, total iridoids, and total polysaccharides at concentrations of 0.91%, 0.52%, 0.66%, and 4.36% (w/w), respectively.

### 2.3. Animal and Treatment

Male ICR mice weighting 20–25 g were obtained from the Laboratory Animal Centre, General Hospital of PLA, Beijing, China. The animals were maintained on a 12 h light/dark cycle under regulated temperature (22 ± 2°C) and humidity (50 ± 10%) and fed with standard diet and water ad libitum. They were allowed to acclimate three days before use. The experimental protocols for the present study have been approved by the Ethics Committee of the PLA General Hospital and were conducted in accordance with the Guide for the Care and Use of Laboratory Animals (China Ministry of Health). All experiments were performed between 09:30–14:00, and each animal was used only once.

The animals were randomly assigned into groups of 50 individuals. Distilled water was given to animals in group 1 (Vehicle group). Animals in group 2 were administered with positive compounds (Desipramine 20 mg/kg). Animals in groups 3, 4, and 5 received intragastric doses of BDT extract powder at 4.5 g, 9 g and 18 g/kg, respectively. The drugs were given daily between 9:30 and 10:30 AM for 14 days. The test was conducted 2 h after the last treatment. The mice, after performing TST behavioral tests, were sacrificed for the determination of monoamine neurotransmitters. 

### 2.4. Forced Swim Test (FST)

The forced swim test was performed according to the method described by Porsolt et al. [21] with modifications. Briefly, mice were forced to swim in a transparent glass vessel (25 cm in high 14 cm in diameter) filled with 10 cm of water at 24 ± 2°C. The total duration of immobility (seconds) was measured as described previously [22] during the last 4 minutes of a single 6-minute test session. Mice were considered immobile when they ceased struggling and remained floating motionless in the water except the movements necessary to keep their heads above the water. 

### 2.5. Tail Suspension Test (TST)

Tail suspension test was carried out according to the method of Steru et al. [23]. Briefly, mice were suspended 5 cm above the floor by means of an adhesive tape placed approximately 1 cm from the tip of the tail. The total duration of immobility (s) was quantified during a test period of 6 minutes. Mice were considered immobile only when they hung passively.

### 2.6. Open-Field Test (OFT)

The ambulatory behaviour was assessed in an open-field test as described previously [24, 25]. The open-field apparatus consisted of a square wooden arena (40 cm × 60 cm × 50 cm) with black surface covering the inside walls. The floor of the wooden arena was divided equally into 12 equal squares marked by black lines. Each mouse was placed individually into the center of the arena and allowed to explore freely. The number of squares crossed by the mouse and the number of rearings on the hind paws were recorded during a test period of 5 minutes. The arena floor was cleaned between the trials with a detergent, and the test was carried out in a temperature-, noise-, and light-controlled room.

### 2.7. Reversal of Reserpine-Induced Ptosis in Mice

The reserpine test was performed according to the method described by Bourin et al. [26] with modifications. Reserpine (2.5 mg/kg) was given intraperitoneally to the animals, and ptosis was evaluated 120 minutes after reserpine treatment. Animals were placed on a shelf (20 cm above the tabletop) and the score of eyes ptosis was calculated as described previously [27], eyes open = 0; one-quarter closed = 1; half closed = 2; three-quarters closed = 3; completely closed = 4.

### 2.8. Measurement of Monoamine Neurotransmitter Levels

To explore the detailed neurochemical mechanisms involved in the antidepressant-like effect of BDT, mice receiving BDT for 14 days were used for the determinations of NE, DA, 5-HT, and 5-HIAA (The metabolite of 5-HT) levels in the brain after TST. Mice were sacrificed by decapitation. Whole brains were rapidly removed from mice, weighted and frozen in liquid nitrogen immediately. The tissue samples were stored at −80°C until assay. Samples were homogenized in 10 volumes of tissue lysis buffer (0.6 mmol/L Perchloric acid, 0.5 mmol/L Na2EDTA and 0.1 g/L L-Cysteine) centrifuged at 15,000 g for 15 minutes. The supernatant was mixed with equal volume of buffer (1.2 mol/L K2HPO4, 2.0 mmol/L Na2EDTA) and centrifuged at 15,000 g for 15 minutes. The resulting supernatant was used for assay. The contents of 5-HT, NA, DA, and 5-HIAA were measured as described previously using high-performance liquid chromatography (HPLC) with fluorescence detection with minor modifications [28]. The supernatant was analyzed by HPLC using an Alltech Alltima C18 column (particle size 5 mm, 4.6 mm × 250 mm). HPLC separation was achieved by an isocratic elution (1 mL/min) with a mobile phase consisting of 87% buffer solution (50 mmol/L citric acid, 50 mmol/L sodium acetate, 0.5 mmol 1-sodium heptanesulfonate, 5 mmol/L triethylamine, and 0.5 mmol/L Na2EDTA, PH = 3.8) and 13% methanol (v/v). The eluate was monitored by fluorescence detector set at emission wavelength 280 nm and excitation wavelength 315 nm. Calibration curve and limit of quantitation were listed (Table 2, Supplementary Material available online at doi:10.1155/2012/419257). The concentration of 5-HT, NE, DA, and 5-HIAA was estimated using a calibration curve of standard solution. The monoamine neurotransmitter levels was expressed as *μ*g/g wet weight of tissue.

### 2.9. Statistical Analysis

The results were expressed as mean ± SEM. Multiple group comparisons were performed using one-way analysis of variance (ANOVA) followed by Dunnett's test in order to detect intergroup differences. A significant difference was determined when *P* < 0.05.

## 3. Results

**Figure 1:**
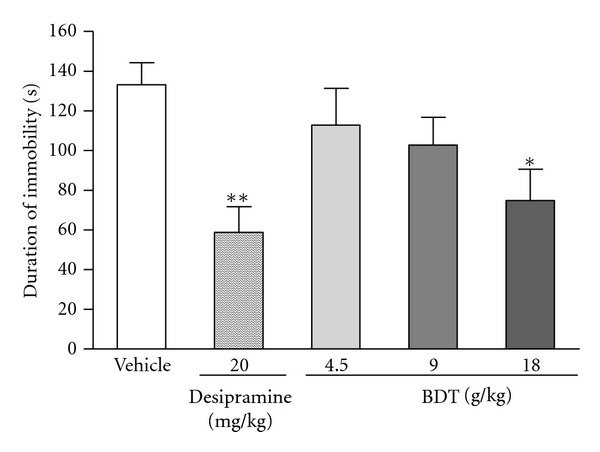
The effect of Baihe Dihuang Tang (BDT, 4.5, 9, 18 g/kg, p.o.) or desipramine (20 mg/kg, p.o.) on the immobility duration of in the forced swimming test. Values given are the mean ± SEM (*n* = 10). *P < 0.05 and **P < 0.01 as compared with vehicle group.

**Figure 2:**
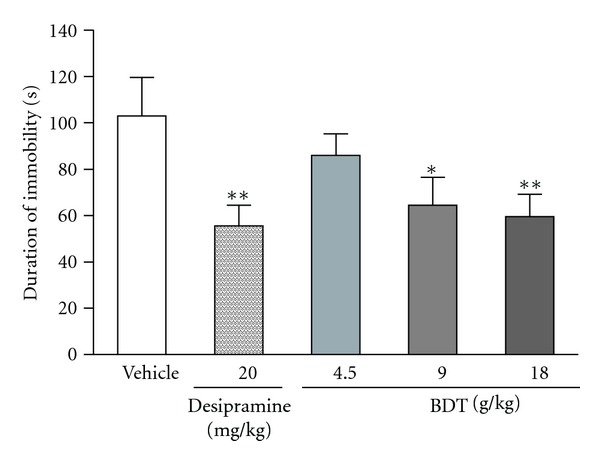
The effect of Baihe Dihuang Tang (BDT, 4.5, 9, 18 g/kg, p.o.) or desipramine (20 mg/kg, p.o.) on the immobility duration of mice in tail suspension test. Values given are the mean ± SEM (*n* = 10). *P < 0.05 and **P < 0.01 as compared with vehicle group.

The FST and TST are the most widely used as behavioural tools for assessing antidepressant activity [29, 30]. The results of BDT on the immobility duration in FST are demonstrated in Figure 1. Compared with the vehicle group, only BDT administration for 14 successive days at dose 18 g/kg decreased the immobility time by 33.8% (*P* < 0.05). The same treatment regimen with BDT at doses of 9 and 18 g/kg also significantly decreased the immobility time in TST. The duration of immobility was reduced respectively by 37.3% and 42.2% when compared with the vehicle (Figure 2). Under the same experimental conditions, the similar effects were observed in mice treated with desipramine at a dose of 20 mg/kg, which served as a positive control of the experiment. The reduction in the duration of immobility for mice given with desipramine was 55.9% and 46.1% in FST and TST, respectively (Figures 1 and 2).

**Figure 3:**
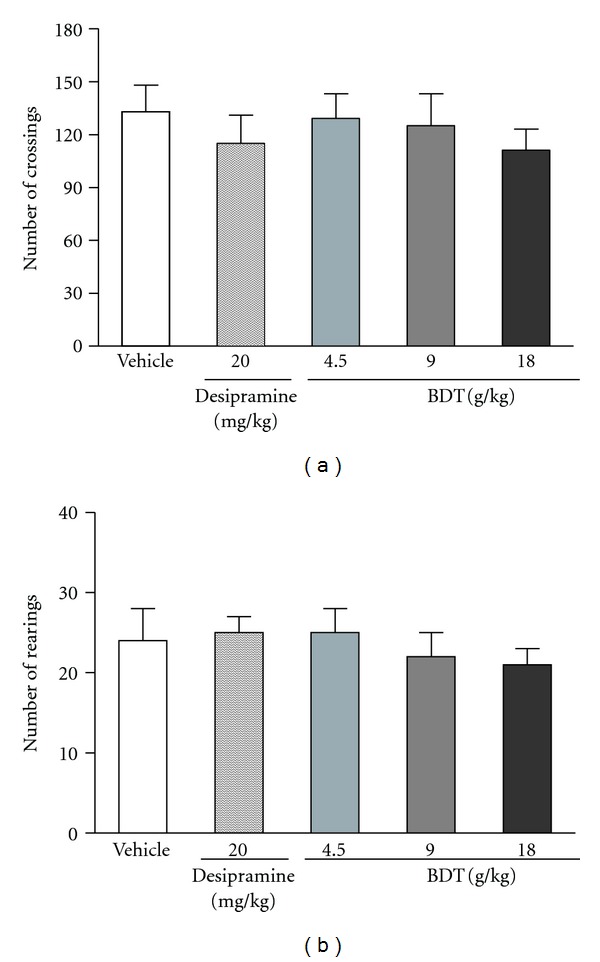
The effect of Baihe Dihuang Tang (BDT, 4.5, 9, 18 g/kg, p.o.) or desipramine (20 mg/kg, p.o.) on the crossings (a) and rearings (b) in the open-field test in mice. Values given are the mean ± SEM (*n* = 10). *P < 0.05 and **P < 0.01 as compared with vehicle group.

As shown in Figure 3, BDT or desipramine administered for 14 successive days did not significantly affect the number of crossings and rearings in the open-field test (OFT) when compared with the vehicle group. It was an indication that the locomotor activity in mice OFT was not affected by the treatment of BDT or desipramine.

**Figure 4:**
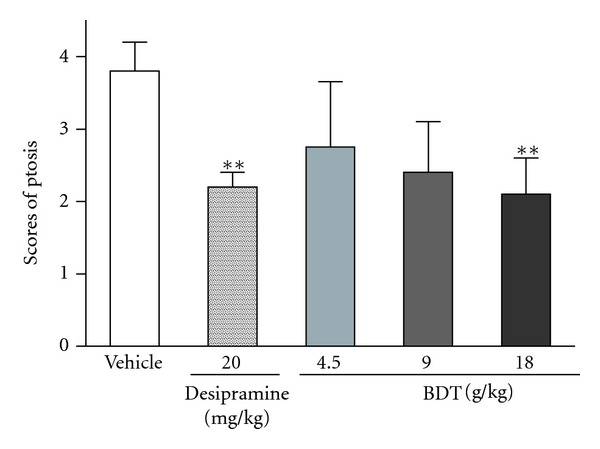
The effect of Baihe Dihuang Tang (BDT, 4.5, 9, 18 g/kg, p.o.) or desipramine (20 mg/kg, p.o.) on reserpine-induced palpebral ptosis in mice. Values given are the mean ± SEM (*n* = 10). *P < 0.05 and **P < 0.01 as compared with vehicle group.

As shown in Figure 4, treating with BDT only at daily doses of 18 g/kg for 14 days significantly antagonized ptosis induced by reserpine. The same treatment protocol using desipramine at 20 mg/kg also significantly antagonized reserpine-induced ptosis.

The data shows BDT has no effect on brain/body of mice (Table 1). The levels of NA, DA, 5-HT, and 5-HIAA in the brain of mice after TST were measured and recorded as shown in Table 1. Compared with normal group, the significantly decreased responses to the TST exposure on 5-HT and NA levels were revealed in mice brain. BDT at 9 and 18 g/kg significantly increased 5-HT levels (*P* < 0.05, *P* < 0.01, resp.), while BDT at 18 g/kg significantly elevated 5-HT metabolite, 5-HIAA, level (*P* < 0.05). NA level was significantly increased after treatment with the higher dose of BDT (18 g/kg). As positive control, desipramine (20 mg/kg) produced an increase in the levels of monoamines 5-HT and NE. No significant changes in DA were observed in any treatment regimen after mice TST. The 5-HT turnover, as represented by the ratio of 5-HIAA/5-HT, was calculated. The significant difference in the 5-HT turnover were observed in group 5 received oral dose at 18 g/kg of BDT (*P* < 0.05) (Table 1).

## 4. Discussion

Forced swim test and tail suspension test are the widely used animal models of depression for the screening of antidepressive activity [21, 23]. In these tests, animals are under stress from which they cannot escape in the confined space. After an initial period of struggling, they would become immobile. Such immobility represented a hopeless state similar to human mental depression and amenable to reversal by antidepressant drugs [21, 23]. In the present study, the antidepressive effects of BDT were assessed by using the two classical animal models. In addition, the effect of BDT on locomotion was evaluated by the OFT for excluding false-positive effects attributable to any psychostimulant effect of BDT. After treated with BDT at 9 and 18 g/kg for 14 days, the mice showed a significant reduction of immobility time in both forced swim (Figure 1) and tail suspension tests (Figure 2). Moreover, BDT treatment did not increase the number of crossings and rearings (Figure 3). Our finding suggested that the reduction of immobility time elicited by BDT treatment in FST and TST was not related to a psychostimulant effect, but rather an antidepressant-like effect of BDT.

Depression has been associated with disturbances of brain monoamine neurotransmitters [31, 32]. As inhibitor, reserpine can irreversibly inhibit the vesicular uptake of monoamines, including noradrenaline, dopamine and 5-hydroxytrytamine and its metabolites. Therefore, we explored the underlying antidepressive mechanism of BDT on the reserpine-induced animal depression model which is based on the monoamine hypothesis of depression [33]. Ptosis is observed as depletion of monoamines reserves or stimulation of monoamines reuptake [34, 35]. In the reserpine-induced ptosis test, the results indicated that antidepressant-like effect of BDT may be involved in the preservation of monoamine neurotransmitters. 

The dysfunction of the central nervous system involving the neurotransmitter 5-HT, NA, and DA has been suggested to play an important role in the pathogenesis of depression. For further evidence for antidepressant-like effect of BDT related to monoamine neurotransmitters, the contents of NE, DA, 5-HT, and 5-HIAA in brain were measured in TST. the TST is commonly used to detect and characterize the efficacy of antidepressant drugs and possesses greater sensitivity than the FST [36]. Our results show that BDT (9 and 18 mg/kg) increased the 5-HT levels in a dose-dependent manner in mice brain. These effects were similar to those observed with the positive drug desipramine. As the ratio of neurotransmitter compared to its metabolites (turnover) can be used as an index of neurotransmitter metabolism, the reduction of turnover indicates a slowdown in the metabolism of neurotransmitters [37]. In present study, the decreased turnover (5-HIAA/5-HT) was observed, indicating a reduction in 5-HT metabolism. Our results suggest that BDT can cause serotonergic activation in the brain, which is consistent with the behavioral changes exhibited in TST. In parallel with the serotonergic system, the noradrenergic system is also important in depression and in mediating behavioral effects of antidepressant drugs [38]. NE level in brain of mice with BDT treatment also showed an increase after TST. The increase is consistent with the effect on reserpine-induced ptosis. Thus, the current study confirmed that the serotonergic system and noradrenergic system might be implicated in the antidepressant-like effect of BDT.

In conclusion, BDT possess antidepressant-like effect in the FST and TST in mice. The results demonstrated that the antidepressant-like effect of BDT is mediated, at least partially, via the central monoaminergic neurotransmitter system.

## Supplementary Material

"Monoamine Neurotransmitter (5-HT, NE, DA, and 5-HIAA) were separated and analyzed by HPLC method. The calibration curve and limit of quantitation were done for estimating the concentration of them in the brain after TST."

## Figures and Tables

**Table 1:** The effect of BDT on the monoamine neurotransmitter levels and 5-HIAA/5-HT turnover ratio in brain after mouse TST.

Group	Dose	Ratio of brain/body (%)	Monoamine neurotransmitter level (*μ*g/g wet tissue)				5-HIAA/5-HT
			NA	DA	5-HT	5-HIAA
Normal	—	1.16 ± 0.024	1.08 ± 0.04	1.21 ± 0.05	1.49 ± 0.04	0.48 ± 0.05	0.32 ± 0.03
Vehicle	—	1.19 ± 0.031	0.77 ± 0.07^##^	0.97 ± 0.09	1.07 ± 0.15^##^	0.35 ± 0.07	0.32 ± 0.02
Desipramine (mg/kg)	20	1.25 ± 0.056	1.15 ± 0.03**	1.01 ± 0.03	1.87 ± 0.05**	0.55 ± 0.03	0.29 ± 0.01
BDT (g/kg)	4.5	1.18 ± 0.025	0.78 ± 0.08	1.04 ± 0.02	1.23 ± 0.13	0.39 ± 0.09	0.31 ± 0.05
	9.0	1.26 ± 0.071	0.79 ± 0.07	1.01 ± 0.05	1.48 ± 0.11*	0.42 ± 0.05	0.28 ± 0.06
	18.0	1.21 ± 0.053	0.91 ± 0.06*	1.02 ± 0.07	1.74 ± 0.06**	0.51 ± 0.04*	0.25 ± 0.02*

Values were expressed as mean ± SEM (*n* = 10). ^##^ *P* < 0.01 as compared with the normal group. *P < 0.05 and **P < 0.01 as compared with the vehicle group.
