# Supplementary material for: Involvement of the Cerebral Monoamine Neurotransmitters System in Antidepressant-Like Effects of a Chinese Herbal Decoction, Baihe Dihuang Tang, in Mice Model
Source: Evid Based Complement Alternat Med. 2012 Aug 23;2012:419257. doi: 10.1155/2012/419257 (PMC3432930; doi:10.1155/2012/419257)
Supplement: Supplementary file 1 — "Monoamine Neurotransmitter (5-HT, NE, DA, and 5-HIAA) were separated and analyzed by HPLC method. The calibration curve and limit of quantitation were done for estimating the concentration of them in the brain after TST." [file 419257.f1.pdf]

Supplementary Material

Table 2. Calibration curve and limit of quantitation (LOQ) for quantitative analysis of five monoamine neurotransmitter in mice brain

Monoamine neurotransmitter Standard compound	calibration curve	R^2	LOQ (μg/ml)
NA	Y=95.47X+18.27	0.9992	0.16
DA	Y=86.58X+14.81	0.9996	0.15
5-HT	Y=824.08X-10.26	0.9993	0.26
5-HIAA	Y=384.95X+11.43	0.9997	0.09
